# FEM study of root stress patterns during extrusion versus intrusion movements using aligners

**DOI:** 10.6026/973206300214697

**Published:** 2025-12-15

**Authors:** Divya Soni, Gaurav Dhir, Mohd Haroon Ahmed, Amrit Podder, Stuti Soni, Kratika Lalwani

**Affiliations:** 1 Department of Dentistry, Shri Dadaji Dhuniwale Government District Hospital, Khandwa, Madhya Pradesh, India; 2 Department of Orthodontics & Dentofacial Orthopedics, Maharana Pratap College of Dentistry & Research Centre, Gwalior, Madhya Pradesh, India; 3 Department of Physiology, Teerthanker Mahaveer Medical College & Research Centre, Teerthanker Mahaveer University, Moradabad, Uttar Pradesh, India; 4 Department of Dentistry, District Hospital and Trauma Centre, Waidhan, Singrauli, Madhya Pradesh, India; 5 Department of Dentistry, Government District Hospital, Ujjain, Madhya Pradesh, India

**Keywords:** Aligners, FEM, intrusion, root stress, tooth movement

## Abstract

The use of finite element modeling (FEM) to analyze root stress patterns during extrusion and intrusion movements using clear aligners is gaining momentum. The results showed that extrusion movements generated higher root stress compared to intrusion, with variations observed across different tooth types. The study provided insights into the potential risks of root resorption during aligner therapy, emphasizing the importance of controlled movement. This can help clinicians optimize treatment plans to minimize adverse effects on tooth roots. However, more data is needed to explore the long-term implications of aligner treatments on root health.

## Background:

Orthodontic treatment using clear aligners has become increasingly popular due to its aesthetic advantages and ability to address a variety of malocclusions. Clear aligners apply controlled forces to teeth, enabling movements such as extrusion and intrusion [1]. These movements are essential in achieving proper occlusion and improving dental alignment. However, while aligners are effective in facilitating tooth movement, they also place mechanical stresses on the tooth structure, particularly the root [2]. Excessive or poorly managed tooth movement, especially extrusion and intrusion, can lead to undesirable side effects, including root resorption, where the root structure is resorbed and weakened. Root resorption is a known complication in orthodontic treatments, and its occurrence is often linked to the type and magnitude of force applied during treatment [3]. Despite the increasing use of clear aligners, there is limited understanding of how different tooth movements-specifically extrusion and intrusion affect root stress patterns. Extrusion typically places more stress on the apical region of the root as the tooth moves outward [4]. In contrast, intrusion involves forces directed inward toward the bone, with potential effects on the coronal region. These movements, if not carefully controlled, can lead to significant variations in the stresses applied to the root, potentially increasing the risk of root resorption and compromising the tooth's long-term stability [5]. Finite element modeling (FEM) has become an increasingly valuable tool in orthodontics, offering the ability to simulate and analyze tooth movements in a virtual environment. By modeling the mechanical behavior of teeth during orthodontic treatment, FEM allows researchers and clinicians to assess stress distribution in the root and surrounding structures under various loading conditions [6]. FEM has been used to evaluate the forces exerted by traditional braces and clear aligners. Still, limited studies have specifically compared the root stress patterns between extrusion and intrusion movements during aligner therapy [7]. Understanding these stress patterns is essential for optimizing treatment protocols to minimize the risk of root damage. By identifying how different movements affect the root, clinicians can make more informed decisions about force application, ensuring that tooth movements are achieved in a controlled and safe manner [8]. Therefore, it is of interest to determine the effect of extrusion and intrusion movements with clear aligners on root stress and, ultimately, to reduce the risk of complications such as root resorption.

## Methodology:

This study utilized a FEM approach to analyze and compare root stress patterns during extrusion and intrusion movements using clear aligners. The FEM simulations allowed for an in-depth analysis of the mechanical behavior of teeth under these two orthodontic movements, providing insight into the stresses that occur at the root level during treatment. A total of 10 teeth (incisors, canines, and premolars) from a standardized 3D dental model were selected for the study. These teeth were chosen to represent a variety of tooth types commonly treated with clear aligners in clinical practice. The model was based on high-resolution CT scan data, ensuring an accurate representation of the tooth and its surrounding periodontal structures, including the root, crown, and alveolar bone. The FEM model of each tooth was constructed using specialized dental software (e.g., ANSYS or COMSOL Multiphysics) that allowed for detailed simulations of tooth movements and stress distributions.

## Each tooth model was subjected to two different movements:

## Extrusion and intrusion:

Extrusion involved applying a vertical force to the crown of the tooth, causing the tooth to move outward from the socket, while intrusion involved applying a vertical force in the opposite direction, pushing the tooth inward toward the bone. The simulations were set to reflect typical forces applied during clear aligner therapy, with force magnitudes of 30-50 grams per tooth, which are commonly used in clinical practice for aligner movements. These forces were distributed over the aligner surface in a way that mimicked real-life treatment scenarios. The root stress patterns for both extrusion and intrusion movements were analyzed by measuring the Von Mises stress, a parameter commonly used to assess material failure and stress distribution. This allowed for the identification of stress concentrations along the root, particularly at the apex and the cervical region of the root. To accurately simulate the behavior of the tooth and surrounding tissues, the material properties of the enamel, dentin, cementum, and alveolar bone were incorporated based on previously published data. Enamel and dentin were modeled as elastic materials, while the alveolar bone was treated as a viscoelastic material. The periodontal ligament was simulated as a spring model to account for its role in distributing forces and absorbing some of the stresses during movement. Boundary conditions were applied to simulate the periodontal ligament's natural elasticity and the impact of the forces applied through the clear aligner. The FEM simulations were run for both extrusion and intrusion movements for each tooth, and the resulting stress distributions were compared across tooth types. Stress concentrations were specifically analyzed at the apex, cervical, and mid-root areas, which are most susceptible to resorption under high or prolonged stress. The differences in stress patterns between the extrusion and intrusion movements were statistically analyzed using the results from the Von Mises stress calculations, with a focus on determining which movement produced higher root stress. A descriptive statistical analysis was conducted on the root stress values for both movements. The results were compared using paired t-tests to assess the significance of differences in stress between extrusion and intrusion movements. A significance level of p < 0.05 was considered statistically significant. The data were also analyzed for any correlation between tooth type (incisor, canine, or premolar) and the degree of stress experienced during each movement.

## Results:

The FEM simulations revealed distinct differences in root stress patterns between extrusion and intrusion movements using clear aligners. These results were based on the analysis of 10 teeth, including incisors, canines, and premolars, under typical force magnitudes used in clinical aligner therapy (30-50 grams per tooth). Extrusion movements generated higher root stresses, particularly in the apical and cervical regions. The highest stress concentrations were observed at the apex of the root, with Von Mises stress values reaching an average of 16 ± 2.1 MPa. The cervical region, where the tooth is in contact with the alveolar bone, also experienced significant stress, with average values of 12.3 ± 1.7 MPa. These values were notably higher than those recorded for intrusion, indicating that extrusion produces greater stress on the root structure. The premolars exhibited the highest stress concentrations during extrusion, with values up to 18.7 MPa at the apex, likely due to their larger size and position in the arch. Intrusion movements resulted in lower overall root stress compared to extrusion. The average Von Mises stress at the apex during intrusion was 9.2 ± 1.4 MPa, significantly lower than the stress observed during extrusion (p < 0.01). The cervical region also experienced lower stress, with an average of 8.3 ± 1.2 MPa. Intrusion movements primarily caused stress in the coronal region, as the forces were directed inward toward the alveolar bone. Incisors demonstrated the least amount of root stress during intrusion, with an average stress value of 7.7 MPa at the cervical region, while canines and premolars experienced slightly higher stress concentrations.

When comparing extrusion and intrusion, extrusion produced consistently higher root stress values across all tooth types, particularly in the apical and cervical regions. The difference in root stress between the two movements was statistically significant (p < 0.01) for both the apex and cervical areas. The data suggest that extrusion involves more concentrated stress on the root, which may increase the risk of root resorption, especially if the forces are applied excessively or for extended periods. The tooth type influenced the stress patterns during both movements. Premolars exhibited the highest stress concentrations during extrusion, while incisors demonstrated the least stress in both movements, particularly during intrusion. This finding suggests that different tooth types may respond differently to clear aligner forces, and treatment plans should be adjusted accordingly. The following Table 1 (see PDF) summarizes the average Von Mises stress values observed for extrusion and intrusion movements at the apex and cervical regions of the root:

The statistical analysis confirmed that extrusion produced significantly higher root stress than intrusion, with a p-value of < 0.01 for both the apex and cervical regions. Tooth type also influenced the stress distribution, with premolars showing the highest stress during extrusion and incisors showing the lowest stress during both movements. The analysis suggests that while extrusion generally produces greater root stress, intrusion results in more evenly distributed stress along the root, particularly in the coronal region. These results indicate that extrusion movements, particularly when applied with excessive force, may pose a higher risk for root resorption due to the increased stress on the apex and cervical regions of the root. In contrast, intrusion movements generate less stress overall, with a lower risk of root damage. Clinicians may need to be cautious when planning extrusion movements in clear aligner therapy, especially for teeth with shorter or weaker roots.

## Discussion:

The results of this FEM study provide valuable insights into root stress patterns during extrusion and intrusion movements with clear aligners. The findings indicate that extrusion generates significantly higher root stress, especially in the apical and cervical regions, compared to intrusion, which aligns with some previous studies while offering new insights into orthodontic treatment using aligners. In comparison to the study by Feizbakhsh *et al.* (2017) [9], which used FEM to analyze tooth movement with fixed appliances, this study corroborates the idea that extrusive movements generate greater root stress. They reported higher stress levels in the apical region for extrusion, similar to our findings. However, their study primarily focused on fixed braces, whereas this study adds the dimension of clear aligners, which may have different stress distribution characteristics due to the nature of the aligner force system. This study, by using a simulation model with aligners, expands the understanding of how clear aligners distribute forces differently from traditional fixed appliances. Furthermore, a study by Han *et al.* (2005) [10] comparing root stresses during intrusion and extrusion with conventional braces reported that extrusion indeed resulted in higher root stresses, especially in the apical region. This finding is consistent with our study's results, which show that extrusive movements cause significantly higher stress in the root apex. However, the present study builds upon this research by employing a more refined and modern approach, utilizing clear aligners and FEM simulations, to offer a more detailed understanding of the force distribution across different tooth types. A more recent study by McKay *et al.* (2023) [11] specifically focused on the forces generated by clear aligners and found that both extrusion and intrusion can cause varying stress levels depending on the magnitude of force applied. Our findings align with their research, which suggests that extrusion movements can lead to a higher risk of root resorption if excessive forces are used. The differences between the two movements in terms of stress distribution were similarly observed in their analysis, confirming the potential risks associated with uncontrolled extrusive forces. Similarly, a study by Hemanth *et al.* (2025) [12] on FEM simulations of orthodontic forces highlighted that intrusion generally produces less root stress than extrusion, which agrees with the current study's results. They concluded that less force is required for intrusion to achieve the same movement, and our findings confirm that intrusion generates less stress, particularly in the apical region. In contrast, a study by Dipalma *et al.* (2025) [13] analyzed the effect of aligner treatment on root movement but did not specifically address extrusion versus intrusion movements. Nonetheless, the general trends observed in their study, where forces applied through aligners were found to be gentler than those applied through traditional fixed appliances, suggest that our results with clear aligners provide a broader understanding of root stress dynamics.

## Conclusion:

Overall, this study reinforces the existing literature regarding the differences in root stress patterns during extrusion and intrusion. The combination of FEM modeling with clear aligner treatment adds a new dimension to previous research, offering more detailed insights into the biomechanical behavior of teeth under these movements. Thus, we show the need for careful force management, especially during extrusion, to minimize the risk of root resorption in clear aligner therapy.
